# A Double Negative Loop Comprising *ETV6/RUNX1* and *MIR181A1* Contributes to Differentiation Block in t(12;21)-Positive Acute Lymphoblastic Leukemia

**DOI:** 10.1371/journal.pone.0142863

**Published:** 2015-11-18

**Authors:** Yung-Li Yang, Ching-Tzu Yen, Chen-Hsueh Pai, Hsuan-Yu Chen, Sung-Liang Yu, Chien-Yu Lin, Chung-Yi Hu, Shiann-Tarng Jou, Dong-Tsamn Lin, Shu-Rung Lin, Shu-Wha Lin

**Affiliations:** 1 Departments of Laboratory Medicine, National Taiwan University Hospital, College of Medicine, National Taiwan University, Taipei, Taiwan; 2 Departments of Pediatrics, National Taiwan University Hospital, College of Medicine, National Taiwan University, Taipei, Taiwan; 3 Departments of Clinical Laboratory Sciences and Medical Biotechnology, College of Medicine, National Taiwan University, Taipei, Taiwan; 4 Institute of Statistical Science, Academia Sinica, Taipei, Taiwan; 5 Department of Bioscience Technology, College of Science, Chung-Yuan Christian University, Taoyuan, Taiwan; 6 Center for Nanotechnology and Center for Biomedical Technology, College of Science, Chung-Yuan Christian University, Taoyuan, Taiwan; Georg Speyer Haus, GERMANY

## Abstract

Childhood acute lymphoblastic leukemia (ALL) with t(12;21), which results in expression of the *ETV6*/*RUNX1* fusion gene, is the most common chromosomal lesion in precursor-B (pre-B) ALL. We identified 17 microRNAs that were downregulated in *ETV6/RUNX1* ^+^ compared with *ETV6/RUNX1* ^−^ clinical samples. Among these microRNAs, miR-181a-1 was the most significantly reduced (by ~75%; P < 0.001). Using chromatin immunoprecipitation, we demonstrated that *ETV6/RUNX1* directly binds the regulatory region of *MIR181A1*, and knockdown of *ETV6*/*RUNX1* increased miR-181a-1 level. We further showed that miR-181a (functional counterpart of miR-181a-1) could target *ETV6/RUNX1* and cause a reduction in the level of the oncoprotein *ETV6/RUNX1*, cell growth arrest, an increase in apoptosis, and induction of cell differentiation in *ETV6/RUNX1* ^*+*^ cell line. Moreover, ectopic expression of miR-181a also resulted in decreased CD10 hyperexpression in *ETV6/RUNX1* ^*+*^ primary patient samples. Taken together, our results demonstrate that *MIR181A1* and *ETV6/RUNX1* regulate each other, and we propose that a double negative loop involving *MIR181A1* and *ETV6/RUNX1* may contribute to *ETV6/RUNX1*-driven arrest of differentiation in pre-B ALL.

## Introduction

The t(12;21) translocation, which fuses *ETV6* and *RUNX1*, is the most common chromosomal alteration in childhood precursor B-cell (pre-B) acute lymphoblastic leukemia (ALL) [1]. The initial fusion of *ETV6/RUNX1* is believed to allow quiescent, preleukemic cells to exist in the bone marrow, and the disease-promoting changes in the *ETV6/RUNX1*-positive preleukemic stage usually take place through second hits that arise in the late pro-B cell stage [2]. The oncogenic property of ETV6/RUNX1 is related to its aberrant function as a rogue transcription factor that can interfere with the normal functions of wild-type ETV6 and RUNX1 through multiple mechanisms. For example, ETV6/RUNX1 can dimerize with wild-type ETV6 via the helix-loop-helix domain of ETV6, thereby disrupting ETV6 function [3, 4]. ETV6/RUNX1 also can bind to RUNX1 target DNA sequences and recruit transcriptional corepressors including mSinA, N-coR, and histone deacetylase-3 (HDAC3) via the ETV6 portion of the fusion protein, resulting in dysregulated RUNX1-dependent transcription [3, 5, 6]. Evidence has revealed that aberrant recruitment of transcriptional repressors correlates with the oncogenic activities of ETV6/RUNX1 so as to constitutively repress a number of genes required for hematopoiesis, including *JunD*, *ACK1*, *PDGFRB*, and *TCF4*, which are involved in cell cycle regulation [7, 8]. The leukemogenic consequences of ETV6/RUNX1 through the aforementioned mechanisms are induction of survival signals and inhibition of cell differentiation by ETV6/RUNX1’s direct modulation of multiple targets such as *EPOR*, *MDM2*, and certain miRNA genes [9–11].

MicroRNAs (miRNAs) execute diverse functions by targeting the mRNAs of multiple genes simultaneously. Recent advances have indicated that miRNAs are important regulators of hematopoiesis; moreover, miRNA-mediated control of gene dosage is critical for lineage fate determination of hematopoietic cells, and disruption of this regulation may lead to malignant transformation [12, 13]. Moreover, dysregulation of miRNA expression is frequently associated with cytogenetic abnormalities, and in turn certain of these abnormalities have a direct impact on aberrant expression of miRNAs [13]. For instance, miR-155 is essential to B-cell development and is aberrantly upregulated in B-cell malignancies including diffuse large B cell lymphoma (DLBCL), follicular lymphoma (FL), and chronic lymphocytic leukemia (CLL) [14–16]. In patients expressing the aberrant fusion protein AML1/ETO, the most common acute myeloid leukemia–associated fusion resulting from t(8;21), the fusion oncoprotein was the first ever reported to directly repress miR-223 expression by triggering chromatin remodeling and epigenetic silencing, which in turn block myeloid precursor cell differentiation [17]. Emerging evidence from research on miRNAs and hematological malignancy has provided deeper insight into the relation between miRNAs and their target genes. In addition to the simple negative regulation of target mRNAs by miRNAs, miRNA–target relationships may also involve complex feedback and feed-forward loops. These loops help to maintain a desired protein inhibition/activation state and often participate in lineage determination in hematopoiesis and lymphomagenesis [12].

It is presumed that ETV6/RUNX1 may occupy the RUNX1-binding motif located in the regulatory regions of certain miRNA genes and thereby disrupt their transcription. Recent studies have shown that aberrant miRNA expression plays an important role in malignant transformation of ETV6/RUNX1 ALL. A highly expressed miR-125b-2 cluster was found in ETV6/RUNX1 ALL, which may provide leukemic cells with a survival advantage against growth inhibitory signals [18]. In addition, downregulation of two miRNAs i.e., miR-494 and miR-320a, by ETV6/RUNX1 via direct binding to the regulatory regions of miRNA genes has been shown to support *ETV6/RUNX1*-positive leukemic cell survival through the loss of inhibition of survivin, an anti-apoptotic protein and the target of miR-494 and miR-320a [11]. Although much is known about these miRNAs and their dysregulation in ETV6/RUNX1 ALL, it remains unclear how and which other miRNAs are involved in ETV6/RUNX1-mediated leukemogenesis.

In an attempt to understand the driving force and the consequence of aberrant miRNA expression in ETV6/RUNX1 ALL, we performed miRNA profiling and lentiviral delivery of miRNAs into pre-B ALL blasts from patients. We identified *MIR-181A1* as the most prominent target of ETV6/RUNX1, and we demonstrate that *ETV6/RUNX1* and *MIR-181A1* form a novel regulatory double negative loop. Our results suggest a mechanism by which ETV6/RUNX1 might exert its preleukemic effect by perturbing the early-stage progression of the B-cell lineage.

## Materials and Methods

### Patients

All of the patient samples were obtained at the time of diagnosis and prior to treatment. The study was approved by the Institutional Review Board of National Taiwan University Hospital. In accordance with the Declaration of Helsinki, we obtained written informed consent from the parents of each patient before collection.

### Cell culture

The REH cells (*ETV6/RUNX1*-positive human pre-B ALL, from ATCC) and human embryonic kidney 293FT cells were cultured in RPMI (Invitrogen) and DMEM (HyClone) medium, respectively, and supplemented with 10% fetal bovine serum (Biological Industries). Human primary pre-B ALL blasts were grown in SFEMII (StemCell Technologies) supplemented with a cytokine cocktail supporting cell growth (StemSpan CC100, StemCell Technologies).

### RNA preparation and gene expression analysis

Total RNA was extracted by Trizol (Invitrogen) and used for reverse transcription (RT) as described [19]. Quantitative real-time PCR (qPCR) was performed on an ABI PRISM 7300. *ETV6/RUNX1* transcripts were detected by TaqMan qPCR using published primer probe combinations [20], and the TaqMan endogenous control assay for *GAPDH* (Applied Biosystems) was used.

MicroRNA expression profiling was performed using the ABI PRISM 7900 and stem-loop RT-qPCR miRNA arrays containing 397 mature human miRNAs (Applied Biosystems) as described [21]. For quantifying individual miRNA each was measured using TaqMan miRNA assays (Applied Biosystems). All miRNA assays were run concurrently with a calibration control, U6 snRNA.

### ChIP

We used the chromatin immunoprecipitation (ChIP) kit (Upstate) to perform the assays. The chromatin was immunoprecipitated with antibodies against RUNX1 and HDAC3 (Abcam). The HDAC inhibitor valproic acid (VPA) was used to release the binding of HDAC3; REH cells were treated with 2 mM VPA for 24 hours before harvesting. Chromatin was also purified from cross-linked DNA that had not been immunoprecipitated to serve as an input control. A genomic region containing the putative RUNX1-binding site located at 3.8 kb upstream of the *MIR181A1* transcription start site (TSS) predicted by CoreBoost_HM (http://rulai.cshl.edu/tools/CoreBoost_HM/) [22], and another *MIR181A1* upstream region which does not contain the RUNX1-binding site were amplified by PCR. As a positive control for RUNX1 ChIP, the primer set PC amplifying the *MIR223* promoter was used as previously described [17]. PCR for the *GAPDH* coding region was carried out as a negative control for HDAC3 ChIP. Primers were listed in Table A in S1 File, available on the *PLOS ONE* Web site.

### Western blotting

Cells were pelleted, washed with cold PBS, and lysed in RIPA buffer (Thermo) with protease inhibitor cocktail (Roche). 35 μg total protein was separated by SDS-PAGE and transferred to an Immobilon PVDF membrane (Pall). The membrane was blocked and incubated overnight with primary antibodies. After a final incubation with secondary antibodies conjugated with horseradish peroxidase (1:5000 dilution; Millipore), immune complexes were detected with HRP chemiluminescent substrate (Millipore). Antibodies and dilutions used were: anti-RUNX1 (1:1000, Abcam) and anti-β-actin (1:5000, Novus).

### Lentiviral construct and infection

The sequence of *MIR181A1* was PCR amplified from human bone marrow mononuclear cells and then cloned into vector pLKO_TRC001 (National RNAi core, Taiwan), which contains a PGK-puromycin acetyltransferase insert, and labeled as pLKO.1.181A1. An empty TRC1 vector, pLKO.1.Null-T (National RNAi Core, Taiwan), which expresses a negative control shRNA (sequence: TCAGTTAACCACTTTTT) was used as an infection control. Production, concentration, and infection of lentivirus followed the protocol from the National RNAi Core, Taiwan. Single infection of REH cells and two sequential infections of primary pre-B ALL blasts with lentiviral particles were carried out. Infected cells were selected by adding puromycin (2 μg/mL) to the culture medium and collected after screening for a week.

### miRNA precursors and siRNA transfection

The two miRNA precursors hsa-mir-181a and negative control 1 (Ambion) are partially double-stranded RNAs that mimic endogenous precursor miRNAs. Each was transfected into cells at a final concentration of 50 nM using siPORT NeoFx transfection agent (Ambion). Two rounds of transfection were performed with a 48-hour interval between the first and second round.

For ETV6/RUNX1 silencing with a short interfering RNA (siRNA), REH cells were transfected with a mixture of siRNAs targeting the fusion region of *ETV6/RUNX1* (Stealth siRNAs, Invitrogen) or a nonfunctional control, siRNA-S (Stealth siRNAs, Invitrogen) [23, 24]. The siRNAs were transfected into REH cells via electroporation with a MP-100 microporator (Labtech) in a 100-μL gold tip under the following conditions: 1 × 10^6^ cells/mL antibiotic-free culture medium, 230 nM siRNA, one pulse of 1,150 V for 30 milliseconds.

### Luciferase reporter assay

The luciferase activity assay was performed using the Dual-Luciferase Reporter Assay System (Promega). A 678-bp fragment of the RUNX1 3′ UTR containing a binding site for miR-181a (UGAAUGU) was cloned into the XbaI site at the distal end of the luciferase reporter gene of pGL3-promoter vector (Promega). This construct was used to transiently transfect 293FT cells with Lipofectamine 2000 (Invitrogen) together with pRL-TK Renilla (Promega), a transfection control used to calibrate the luciferase activity, and pLKO.1.181A1 (miR-181a-expressing vector) or pLKO.1.Null-T (negative control for miR-181a-expressing vector). A mutated version of the binding sequence (AGAUCUG) containing a BglⅡsite was used as the target site control. Cells were lysed, and the luciferase activity was measured 48 hours after transfection.

### Cell viability, cell cycle, proliferation, and apoptosis assays

The cell viability was determined by Cell Proliferation Kit I (MTT) (Roche). A BrdU flow kit (BD) was used to determine cell cycle and proliferating cells. Apoptosis was evaluated by annexin V: FITC apoptosis detection kit (BD).

### Flow cytometry analysis of lineage markers

Monoclonal antibodies recognizing the following cell-surface markers were used for flow cytometry: CD10, CD19, CD20, CD45, IgM, κ-chain, and λ-chain (BD). Flow cytometry was performed using FACScalibur (BD). Data were analyzed using FCS Express software (De Novo Software).

### Statistical analyses

In miRNA profiling analysis, to avoid low abundant expression issue, miRNA with coefficient of variation (CV) < 0.2 was removed in the first step. In the second step, the student’s t test was used to evaluate different miRNA expression between *ETV6/RUNX1*-positive (n = 10) and *ETV6/RUNX1*-negative (n = 40) groups. Finally, in order to control multiple testing issues, false discovery rate method was performed to adjust p value obtained from student’s t test [25]. Data are represented the means ± SE or ± SD as indicated in the figure legends. The Student’s t test or ANOVA were used to test the difference between groups for continuous variables. For categorical data, Fisher’s exact was performed to test the difference between groups. Calculation methods of P values were denoted in the figure legends or bottom of tables. All tests were two-tailed and P values <0.05 were considered significant.

## Results

### miR-181a-1 is downregulated in *ETV6/RUNX1*-positive leukemias, and the regulatory region of *MIR181A1* is bound by *ETV6/RUNX1* and HDAC3

**Fig 1:**
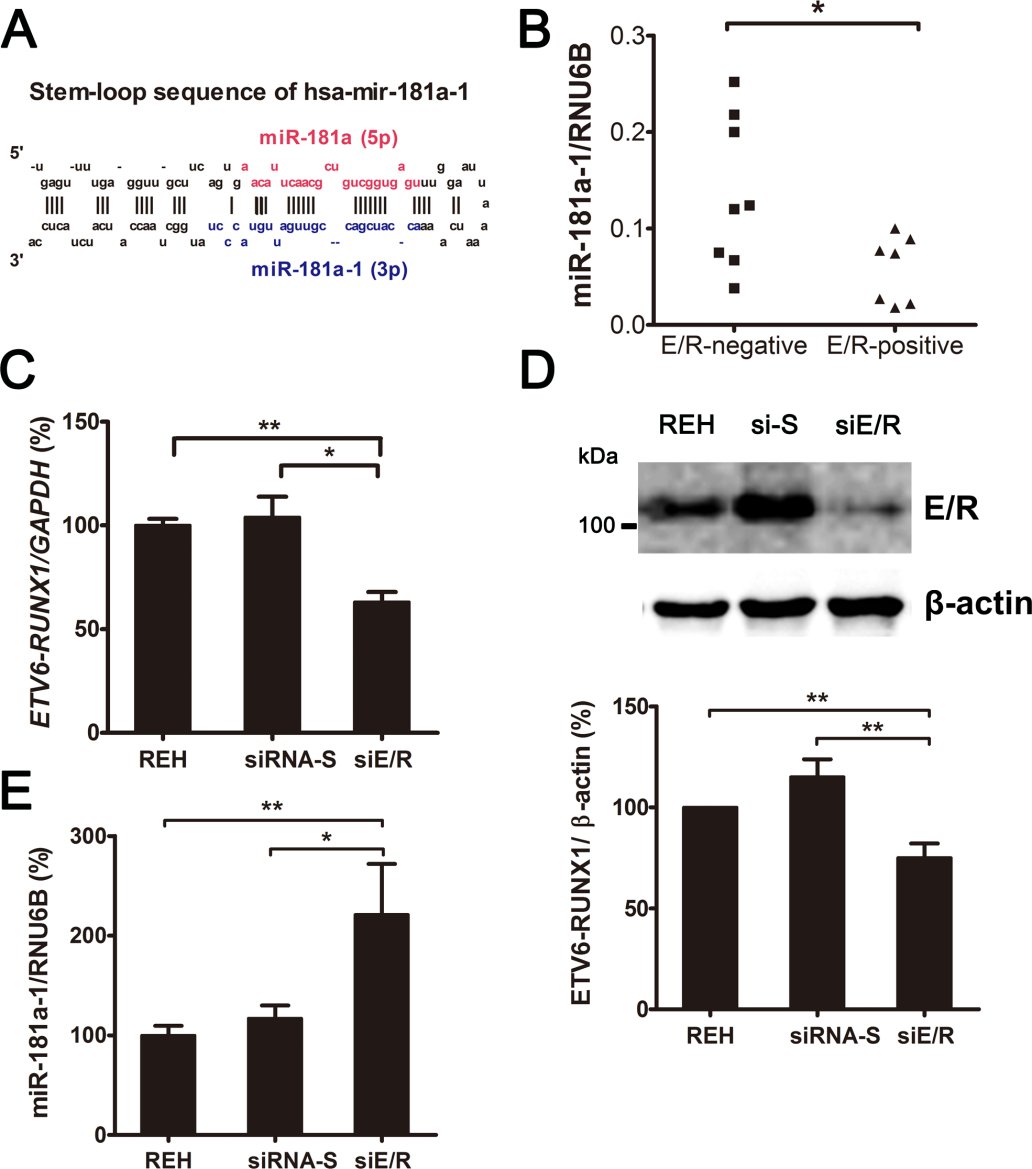
siRNA-mediated silencing of *ETV6/RUNX1* increases the level of mature miR-181a-1. (A) Stem-loop sequence of human precursor mir-181a-1. Mature miR-181a (red) and miR181a-1 (blue) are indicated. (B) Validation of miR-181a-1 expression in primary pre-B ALL samples (*ETV6/RUNX1*-positive, n = 7; *ETV6/RUNX1*-negative, n = 8) using TaqMan qRT-PCR. ETV6/RUNX1-expressing REH cells (control) were transfected with siRNAs. After 48 hours of transfection with functional siETV6/RUNX1 (siE/R) or nonfunctional siRNA (siRNA-S), (C) *ETV6/RUNX1* mRNA was detected by qRT-PCR, and (D) protein was analyzed by Western blotting with anti-RUNX1; anti-β-actin was used as a loading control. Relative expression as determined by densitometry is indicated below the blots. (E) Mature miR-181a-1 was measured by qRT-PCR. Bars represent the mean ± SE of at least three independent experiments. *GAPDH* and *RNU6B* were used as calibration controls for mRNA and miRNA expression, respectively. *P ≤ 0.05, **P ≤ 0.01 (ANOVA).

Extensive miRNA profiling was carried out on the diagnostic samples of a cohort of 50 pre-B ALL patients, including 10 *ETV6/RUNX1*-positive and 40 *ETV6/RUNX1*-negative cases (clinical feature of patients see Table B in S1 File). Because ETV6/RUNX1 retains the DNA-binding ability of RUNX1, the fusion protein acts as a dominant-negative repressor to downregulate RUNX1 target genes. Therefore, a reduction of specific miRNAs in *ETV6/RUNX1*-positive samples compared with *ETV6/RUNX1*-negative samples was evaluated. Seventeen miRNAs were significantly downregulated in *ETV6/RUNX1*-positive ALL samples (Table 1), and of these, miR-181a-1, which is derived from the 3′ arm of precursor hsa-mir-181a-1 (Fig 1A), had the most significant *P*-value and showed a remarkable 4-fold decrease (Table 1). The decreased expression of miR-181a-1 in *ETV6/RUNX1*-positive leukemias was validated in another cohort of pre-B ALL cases analyzed by real-time qRT-PCR (Fig 1B).

**Table 1:** The statistic signature of 17 miRNAs.

miRNA*	Expression level,	*P*
	ETV6/RUNX1^+^/ ETV6/RUNX1^-^	
hsa-miR-181a-1	0.254	< .00005
hsa-miR-92	0.327	.002
hsa-miR-222	0.194	.004
hsa-miR-342	0.461	.004
hsa-miR-181d	0.524	.004
hsa-miR-155	0.353	.005
hsa-miR-423	0.371	.005
hsa-miR-195	0.391	.012
hsa-miR-130b	0.472	.019
hsa-miR-221	0.098	.024
hsa-let-7b	0.505	.037
hsa-let-7a	0.527	.037
hsa-miR-30e-3p	0.443	.039
hsa-miR-19a	0.456	.039
hsa-miR-660	0.525	.045
hsa-miR-181c	0.385	.046
hsa-miR-425	0.465	.050

* Selected by differential expression in patients with or without *ETV6/RUNX1* fusion gene.

Whether ETV6/RUNX1 regulates miR-181a-1 expression was further assessed by siRNA-mediated knockdown of *ETV6/RUNX1* in REH cells, which express the ETV6/RUNX1 fusion protein (Fig 1C and 1D). A mixture of two *ETV6/RUNX1*-specific siRNAs (siE/R), which target the fusion region of *ETV6/RUNX1*, was used to suppress *ETV6/RUNX1* expression [23]. As a transfection control, we used a nonfunctional siRNA (siRNA-S) that had no effect on *ETV6/RUNX1* expression [24]. Compared with siRNA-S, both mRNA and protein of ETV6/RUNX1 were significantly reduced by ~40% and ~35% after knockdown with siE/R (Fig 1C and 1D). Further examination showed that miR-181a-1 levels increased significantly in REH cells that were treated with siE/R but not in those treated with siRNA-S (Fig 1E). These studies with REH cells and clinical leukemic specimens indicated that ETV6/RUNX1 negatively regulates miR-181a-1 level.

**Fig 2:**
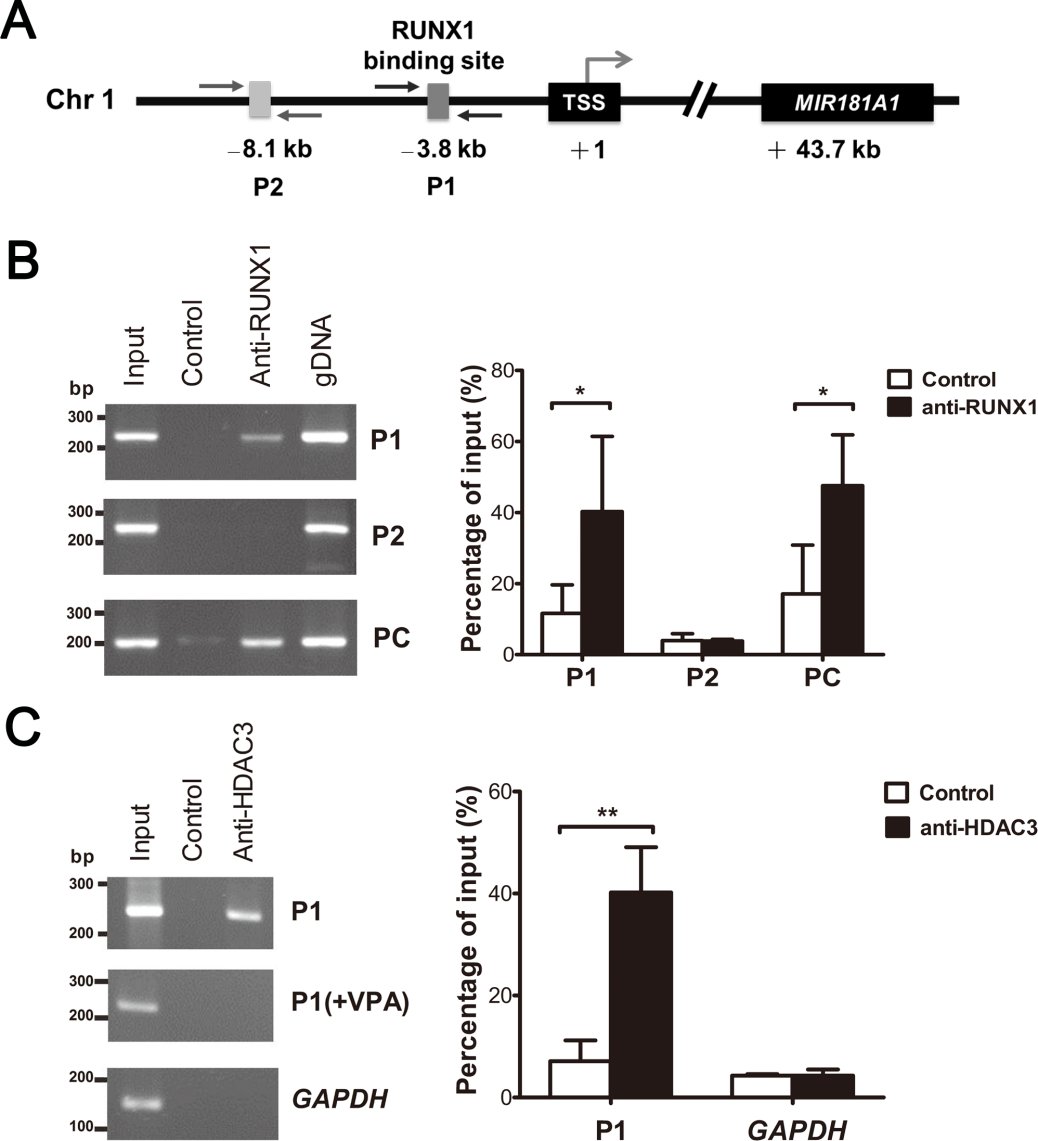
*ETV6/RUNX1* inhibits *MIR181A1* transcription via binding to the endogenous RUNX1 site. (A) Schematic representation of the genomic structure of human *MIR181A1*. The location of the *MIR181A1* gene and the RUNX1-binding site are numbered relative to the TSS (+1). Arrows indicate the locations of the primers used in the ChIP assay. (B) ChIP was carried out using anti-RUNX1 or in the absence of specific antibody (Control) (left). DNA sequences surrounding the putative RUNX1-binding site were amplified by PCR using P1 primers. To evaluate the specificity of RUNX1 binding, a positive control and a negative control were performed using PC and P2, respectively, for the ChIP assay. Amplification of the upstream region near the RUNX1-binding site on *MIR223*, which is a known direct target of RUNX1, was performed using PC primers. P2 primers were designed to amplify a distal region lacking the RUNX1-binding site. Input shows the amplification from sonicated chromatin, and genomic DNA (gDNA) was used as a positive PCR control. The PCR products were quantified by densitometry (right). (C) The ChIP assay was performed using anti-HDAC3 (left). Treatment with valproic acid and amplification of the promoter region of *GAPDH* were used as controls. The PCR products were quantified by densitometry (right). Bars show the mean ± SD from three independent experiments. *P ≤ 0.05, **P ≤ 0.01 (ANOVA).

To reveal the interaction between ETV6/RUNX1 and the regulatory region of *MIR181A1*, we performed ChIP using the REH cells and a RUNX1-specific antibody. Bioinformatic analyses identified the predicted transcription start site (TSS) of *MIR181A1* and a putative RUNX1-binding site with the sequence of TGT/cGGT located 3.8 kb upstream of the TSS (P1 site, Fig 2A; Table C in S1 File). Binding of RUNX1 and ETV6/RUNX1 at P1 was demonstrated by specific precipitation of this DNA region, but not at an irrelevant site (P2), with anti-RUNX1 in the ChIP analysis (Fig 2B). Moreover, ChIP with anti-HDAC3 also revealed the binding of HDAC3 at P1 (Fig 2C). These results are in agreement with previous reports that the transcriptional repressor activity of ETV6/RUNX1 is associated with its aberrant recruitment of the N-CoR/SMRT-HDAC3 complex [5–7]. Taken together, these results supported the idea that ETV6/RUNX1 directly regulates *MIR181A1* expression.

### miR-181a targets *ETV6*/*RUNX1*

**Fig 3:**
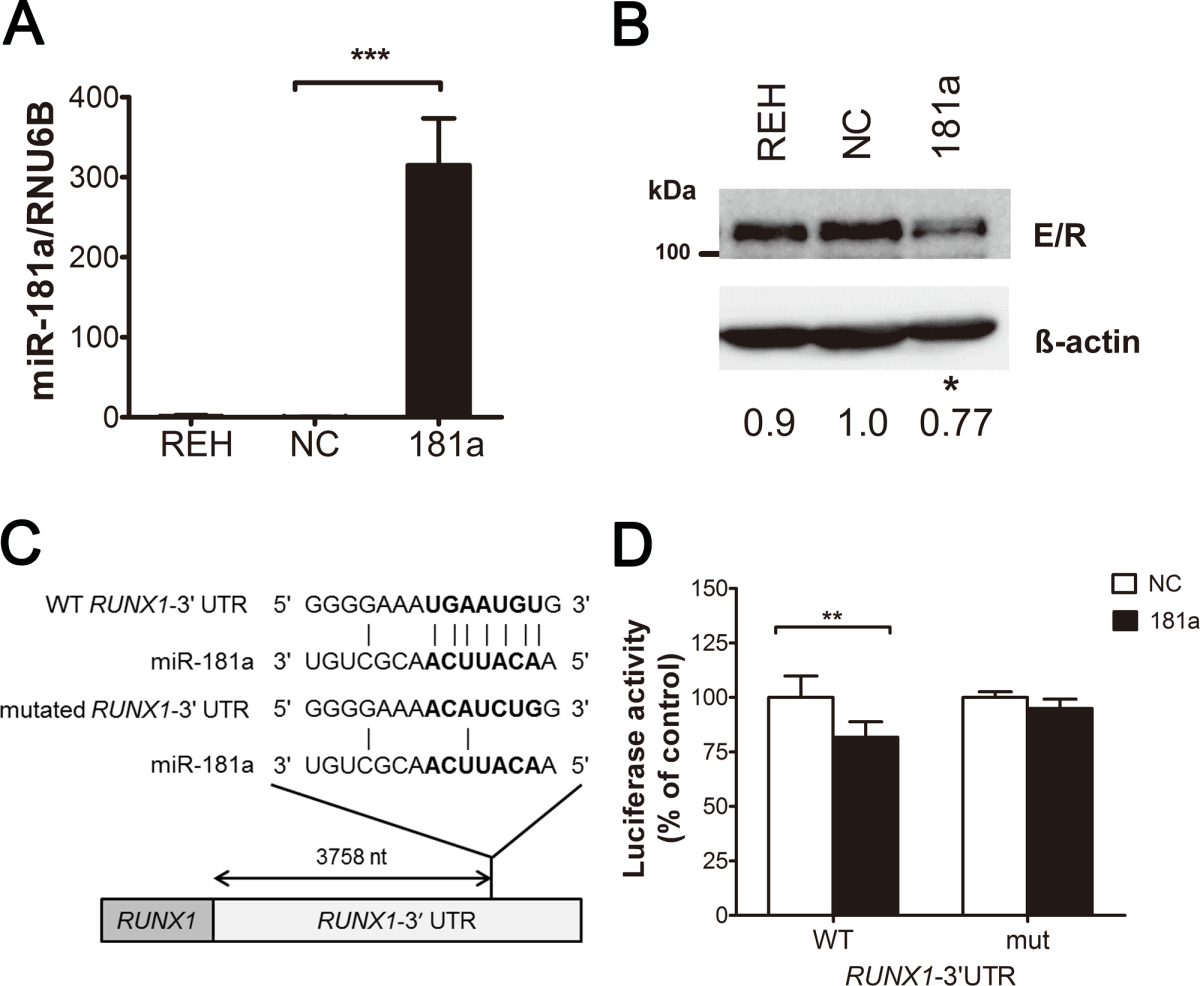
miR-181a regulates the level of *ETV6/RUNX1*. Overexpression of miR-181a in REH cells was performed by transfection with precursor miRNA. A final 50 nM concentration of nontargeting-miR (NC) or pre-mir-181a (181a) were transfected twice and cells were harvested after 48 hours of the second transfection for further examination. (A) miR-181a level was detected by TaqMan qRT-PCR. (B) Regulation of ETV6/RUNX1 by miR-181a was confirmed by Western blotting with RUNX1-specific (E/R) antibody. Relative expression as determined by densitometry is indicated below the blots. (C) The putative miR-181a binding site in the *RUNX1* 3' UTR. nt, nucleotides. (D) The last 678 bp of the human *RUNX1* 3' UTR containing normal (WT) or mutated (mut) miR-181a targeting sequences were cloned downstream of a pGL3-luciferase vector and transfected into 293FT cells with expression vectors for miR-181a (181a) or negative control shRNA (NC). All experiment was conducted in triplicate. Bars represent the mean ± SD of three independent experiments. *P ≤ 0.05, **P ≤ 0.01, ***P ≤ 0.001 (ANOVA).

The consequence and mechanism(s) of *MIR181A1* downregulation in ETV6/RUNX1 ALL were further investigated. It has been shown that miR-181a, a mature form derived from the 5′ arm of precursor hsa-mir-181a-1 (Fig 1A), functions by targeting several mRNAs [26–28]. To identify new miR-181a target genes, we conducted a database search utilizing TargetScan (http://www.targetscan.org), an online miRNA target prediction interface, and searched for oncogenes targeting by miR-181a. With miRNA target prediction programs, we identified 1,194 potential miR-181a target genes. The presence of *RUNX1* among the database-predicted target genes implies an unknown mechanism of *ETV6/RUNX1* regulation by miR-181a. To investigate this hypothesis, we first overexpressed miR-181a in REH cells by transfection of miRNA mimics (Fig 3A), which resulted in a decrease of ETV6/RUNX1 (Fig 3B). The negative effect of miR-181a on *ETV6/RUNX1* expression was further assessed with the luciferase reporter assay, which examined the interaction between miR-181a and the 3' UTR of *RUNX1* and *ETV6*/*RUNX1* (Fig 3C). We constructed fragments containing the last 678 bp of the *RUNX1* 3′ UTR, which contains wild-type or mutated miR-181a recognition sequence, and inserted them immediately downstream of the luciferase reporter gene. The miR-181a expression vector or empty vector was co-transfected with the different luciferase 3' UTR constructs into 293FT cells. The results showed that miR-181a downregulated the luciferase reporter gene activity when the luciferase gene was fused with wild-type but not mutated *RUNX1* 3' UTR (Fig 3D). These experiments demonstrated that miR-181a targets *ETV6/RUNX1*, and they suggested that the fusion gene and *MIR181A1* can regulate each other.

### Ectopic expression of miR-181a in REH cells impedes cell growth and enhances cell differentiation

**Fig 4:**
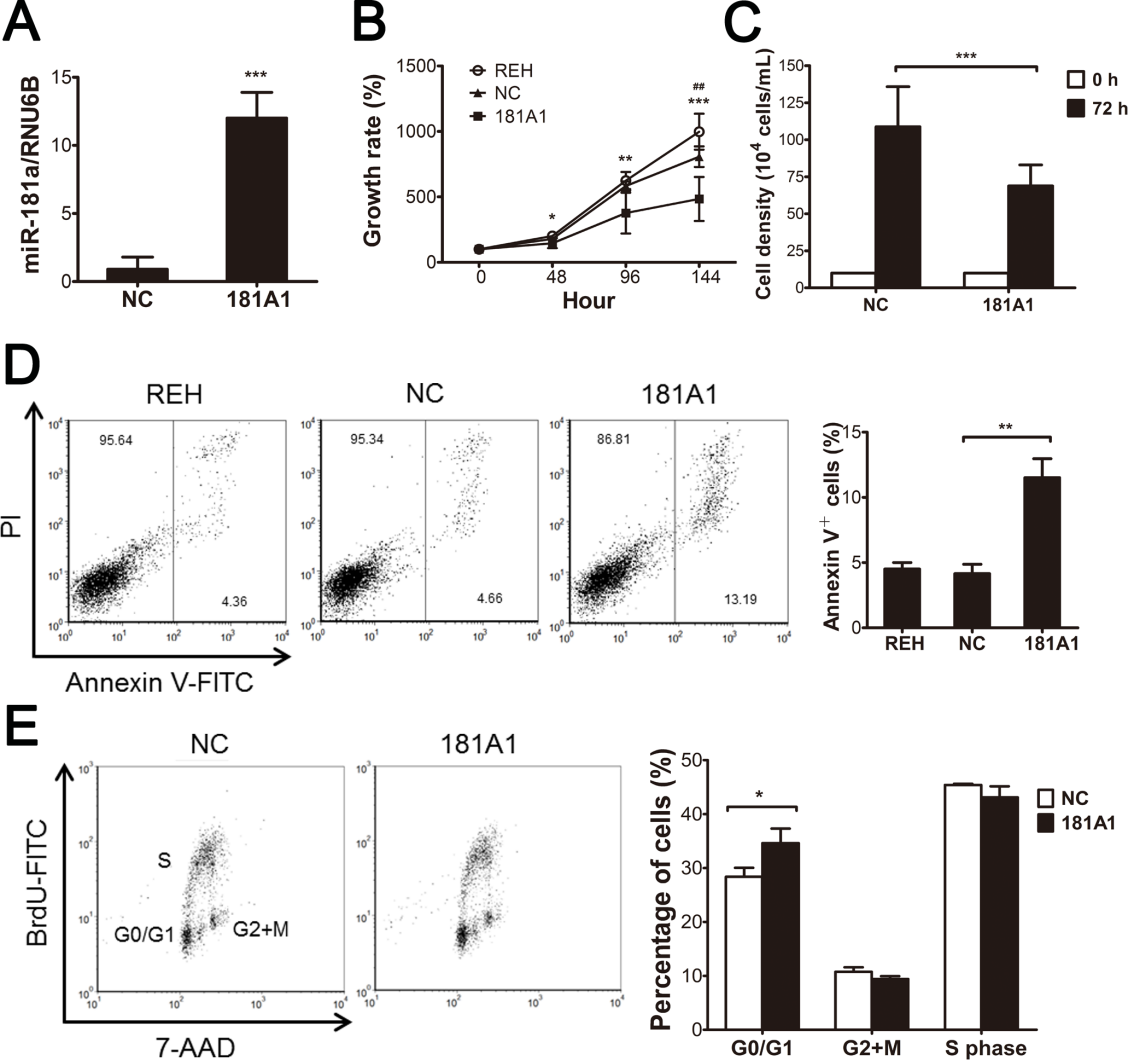
Ectopic expression of miR-181a suppresses growth and induces apoptosis of REH cells. REH cells were infected with lentiviral vector expressing the negative control shRNA (NC) or miR-181a (181A1). (A) Relative miR-181a levels were determined by qRT-PCR. (B) Growth curve was determined as following: Cells were cultured for 48, 96, and 144 hours and then assessed with the MTT assay. (C) Cells were seeded at a density of 1 × 10^5^ cells/mL and cultured. After 72 hours, cells were stained with trypan blue, and viable cells were counted. (D) Apoptosis was assessed by flow cytometric analysis of annexin V/propidium iodide (PI) staining of lentivirus-transduced cells (left). Representative histograms demonstrate the proportion of annexin V-positive cells (right). (E) Biparametric BrdU/DNA analysis: During the last 30 minutes of culture, 1 mM BrdU was added to the cells, and then the cells were stained with anti-BrdU and 7-aminoactinomycin D (7-AAD) and detected by flow cytometry (left). The percentage of cells in each of the cell-cycle phases G0/G1, S, and G2+M was quantified (right). Bars show the mean ± SD from three independent experiments. 181A1 vs. NC *P ≤ 0.05, **P ≤ 0.01, ***P ≤ 0.001 (ANOVA); NC vs. REH ^##^ *P* ≤ 0.01.

The oncogenic effect of ETV6/RUNX1 has been postulated to operate through impairment of B-cell differentiation in a bone marrow transplantation model, and consequently it results in the accumulation of pro-B cells [29]. We investigated whether the greatly reduced *MIR181A1* expression in *ETV6/RUNX1*-positive pre-B ALL blasts plays a role in the ETV6/RUNX1-mediated blockade of B-cell differentiation and in the preleukemic events induced by ETV6/RUNX1. First, REH cells were transduced by a lentiviral vector carrying *MIR181A1* (181A1-LV) to express miR-181a stably and constitutively (Fig 4A). We found that ectopic overexpression of miR-181a resulted in growth retardation of the cells, and 181A1-LV-transduced REH cells showed a nearly 40% decrease in both MTT activity and cell density after 72 hours of seeding (Fig 4B and 4C). Moreover, 181A1-LV transduction increased the annexin V–positive (apoptotic) cell population (Fig 4D). Further assessment of the proliferation activity by biparametric BrdU/DNA flow cytometry showed that miR-181a expression did not affect the percentage of BrdU-positive cells but rather increased the proportion of cells in G0/G1 phase (Fig 4E).

**Fig 5:**
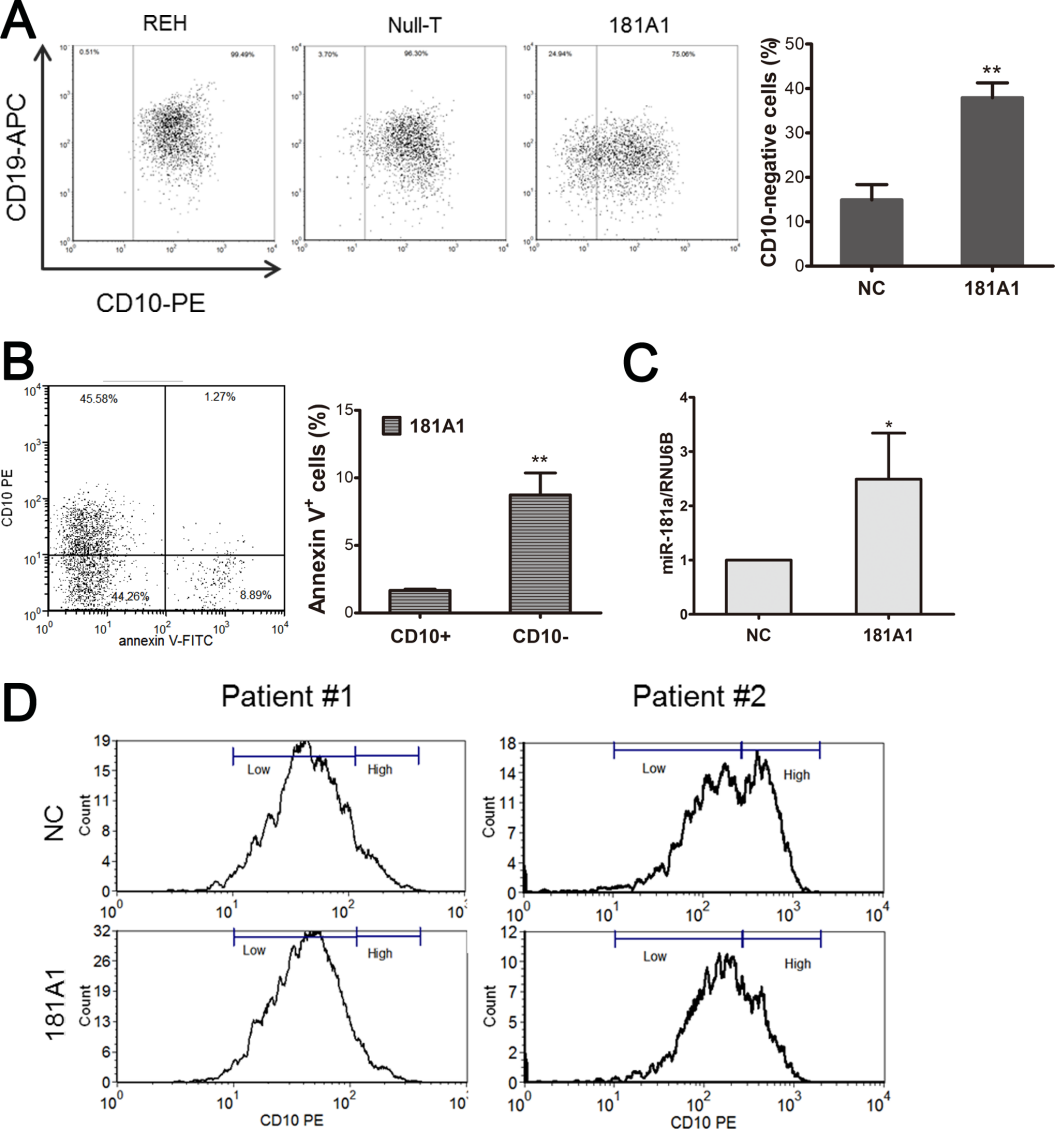
Ectopic expression of miR-181a enhances lymphoid differentiation in the *ETV6/RUNX1*-positive cell line and primary ALL blasts. (A) Percentage of lentivirus-infected REH cells stained for cell-surface marker CD10 as analyzed by flow cytometry (left). The results were quantified and are presented as the average ± SD of three independent evaluations (right). (B) 181A1-LV-infected REH cells were co-stained for annexin V and CD10 and measured by flow cytometry (left). The percentage of each of CD10-positive and -negative annexin V-positive cells was quantified, and the average ± SD of three independent evaluations is shown (right). (C) Primary ALL blasts were infected with NC- or 181A1-LV. Mature miR-181a expression level in lentivirus-infected primary pre-B ALL blasts from three ETV6/RUNX1-positive patients was determined by qRT-PCR. The results are shown as average ± SD. (D) CD10 expression in CD45^w+or+^ CD19^+^ ETV6/RUNX1-positive pre-B ALL blasts was analyzed by flow cytometry. In the comparison with the infection control (NC), 181A1-LV-transduced cells derived from patient #1 and #2 both showed a decrease in CD10^High^ cells (Patient #1: NC 10.1% / MFI 40.92, 181A1 6.95% / MFI 39.51; Patient #2: NC 40.1% / MFI 424.28, 181A1 29.5% / MFI 444.24) and a relative increase in CD10^Low^ cells (Patient #1: NC 87.98% / MFI 165.49, 181A1 91.05% / MFI 156.68; Patient #2: NC 58.1% / MFI 1929.88, 181A1 69% / 1854.4). *P ≤ 0.05, **P ≤ 0.01 (ANOVA).

The stages of B cell maturation are characterized by specific expression patterns of immunoglobulins and other membrane proteins. To gain insight into the effect of miR-181a overexpression on REH cell maturation, we stained cells for differentiation markers and found an increase in CD10-negative, CD20-positive, surface IgM-positive, κ-chain-positive, and λ-chain-positive cell populations in 181A1-LV-transduced cells compared with infection control cells (Fig 5A and S1 Fig). Decreased CD10 expression and increased CD20, IgM, κ-chain, and λ-chain expression may represent a gradual progression of B lymphoid cells from pre-BI cells to immature B cells [30]. Because the decrease in CD10 expression was the most notable change of 181A1-LV-transduced cells, we further stained cells for CD10 and annexin V and found that most apoptotic cells were CD10-negative (Fig 5B).

### miR-181a induces partial differentiation by diminishing CD10 expression in *ETV6/RUNX1*-positive pre-B ALL blasts

Loss of the marker CD10 and a gain of CD20 have been associated with differentiation of normal B-cell precursors from hematopoietic stem cells to naive mature B cell in the bone marrow [31]. The infection of primary blasts isolated from the bone marrow of pre-B ALL patients with a lentiviral vector expressing miR-181a increased the level of miR-181a by an average of 2.5-fold (range from 1.5- to 3-fold) in three *ETV6/RUNX1*-positive samples compared with the controls (Fig 5C). This induction partially altered the lymphocytic differentiation as shown by the CD10 hyperexpression decrease in cells from two of three *ETV6/RUNX1*-positive samples (Fig 5D and S2 Fig), suggesting that the level of miR-181a expression is important for the perturbation of the lymphocytic differentiation program in ETV6/RUNX1 ALL.

## Discussion

The critical roles of miRNAs in hematopoiesis and their ubiquitous dysregulation in leukemia allow us an opportunity to understand the driving force of leukemogenesis and the consequences of aberrant miRNA expression. By applying miRNA profiling to samples from 50 pre-B ALL patients, we determined the gene expression signatures of specific ALL subtypes. The miRNA expression profile showed that most miRNAs are downregulated in *ETV6/RUNX1*-positive samples, indicating that ETV6/RUNX1 affects the functions of miRNAs primarily by downregulating their expression. We identified miR-181a-1 as the most differentially underexpressed miRNA in patients carrying t(12;21). This is consistent with the expression profile of another patient cohort; Schotte et al. measured 397 miRNAs in 81 pediatric ALL cases and also demonstrated that miR-181a-1 expression is 5-fold lower in patients with t(12;21) than in patients with other ALL subtypes [32]. To address how ETV6/RUNX1 regulates miR-181a-1 level, we performed siRNA-mediated knockdown of *ETV6/RUNX1* followed by ChIP in an ETV6/RUNX1-expressing leukemic cell line. Our data reveal the upregulation of miR-181a-1 in ETV6/RUNX1-knockdown cells and direct ETV6/RUNX1 binding and recruitment of HDAC3 to the regulatory region of *MIR181A1*, suggesting that ETV6/RUNX1 negatively regulates *MIR181A1* expression.

The miR-181 family is highly conserved and comprises six miRNAs transcribed from three separate gene loci and organized into three clusters including miR-181a/b-1, miR-181a/b-2, and miR-181c/d. The finding that both miR-181a-1 and miR-181c/d are significantly downregulated in ETV6/RUNX1 ALL (Table 1) and that all members of the miR-181 family share the same seed sequence within their 5′ arms and in targets led us to investigate which downstream genes are regulated by miR-181a. We found not only was miR-181a-1 suppressed by ETV6/RUNX1, but feedback inhibition of miR-181a on ETV6/RUNX1 was observed in the cell line experiments, suggesting that *MIR181A1* and *ETV6/RUNX1* can regulate each other.

In fact, such a regulatory network between transcription factors and miRNAs has been described before; for example, regulatory circuitry comprising miR-223 and transcription factors NFI-A and C/EBPα has been shown to sustain the level of miR-223, which may be important in granulopoiesis [33]. Moreover, recently a ‘mutual negative feedback loop’ involving MYC and miR-548m was described in non-Hodgkin B-cell lymphomas, in that this regulatory loop is important for sustaining a high level of MYC and low level of miR-548m during lymphomagenesis and drug resistance [34]. According to previous findings and our current data, we propose a new mechanism of ETV6/RUNX1 action: a double negative loop in which ETV6/RUNX1 can bind to the regulatory region of *MIR181A1* keeps hsa-mir-181a-1 expression low, which consequently reduces the miR-181a-mediated translational repression of ETV6/RUNX1. By doing so, ETV6/RUNX1 can enhance its own oncogenic potential (S3 Fig).

In conclusion, our study enhances the understanding of the molecular mechanism underlying ETV6/RUNX1-mediated attenuation of B-cell differentiation and offers the opportunity to identify new targets for development of therapeutic approaches to leukemia.

## Supporting Information

S1 Fig Surface antigen of lymphoid lineage expressed on lentivirus-infected REH cells. Expression of (A) CD20, (B) IgM, (C) κ-chain, and (D) λ-chain were detected by flow cytometric analysis (left). The results were quantified and represented as the average of three independent evaluations ± SD (right). *P ≤ 0.05, **P ≤ 0.01, ***P ≤ 0.001 (ANOVA).
(TIF)

S2 Fig Surface CD10 expression of lentivirus infected CD45^+^ CD19^+^ *ETV6/RUNX1*-positive pre-B ALL blasts. Flow cytometric analysis of CD10 expression on CD45^w+^or+ CD19^+^ *ETV6/RUNX1*-positive pre-B ALL blasts derived from patient #3. The MFI of CD10 in each groups were: NC 49.62, 181A1 51.68.
(TIF)

S3 Fig Schematic representation of the reciprocal downregulation of *ETV6/RUNX1* and *MIR181A1*. In leukemia cells with frequent chromosome rearrangement t(12;21)(p13;q22), ETV6/RUNX1 oncoprotein occupies the putative RUNX1-binding site upstream of *MIR181A1* and restricts transcription by recruiting co-repressors such as HDAC3. This repression of *MIR181A1* expression consequently upregulates the target of miR-181a, ETV6/RUNX1, the oncoprotein itself.
(TIF)

S1 File Primer sequences (Table A). Clinical features of the ALL patients included in expression profiling study (Table B). The signature of 13 miRNAs/miRNA cluster and the location of RUNX1 binding sites (Table C). (DOCX)
