# Supplementary figures and images for: A Double Negative Loop Comprising ETV6/RUNX1 and MIR181A1 Contributes to Differentiation Block in t(12;21)-Positive Acute Lymphoblastic Leukemia
Source: PLoS One. 2015 Nov 18;10(11):e0142863. doi: 10.1371/journal.pone.0142863 (PMC4651427; doi:10.1371/journal.pone.0142863)

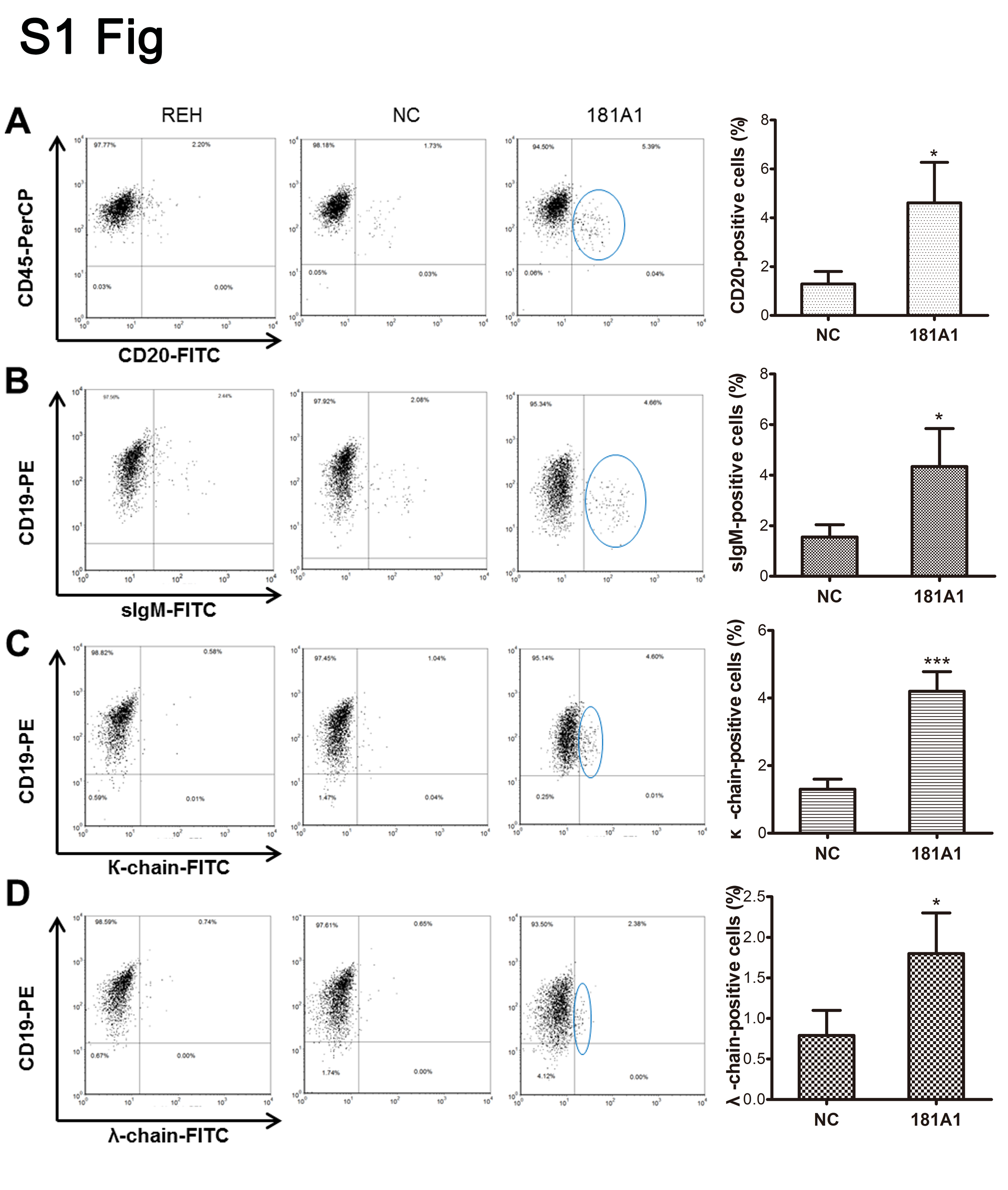

Supplement: S1 Fig — Expression of (A) CD20, (B) IgM, (C) κ-chain, and (D) λ-chain were detected by flow cytometric analysis (left). The results were quantified and represented as the average of three independent evaluations ± SD (right). *P ≤ 0.05, **P ≤ 0.01, ***P ≤ 0.001 (ANOVA). (TIF) [file pone.0142863.s001.tif]

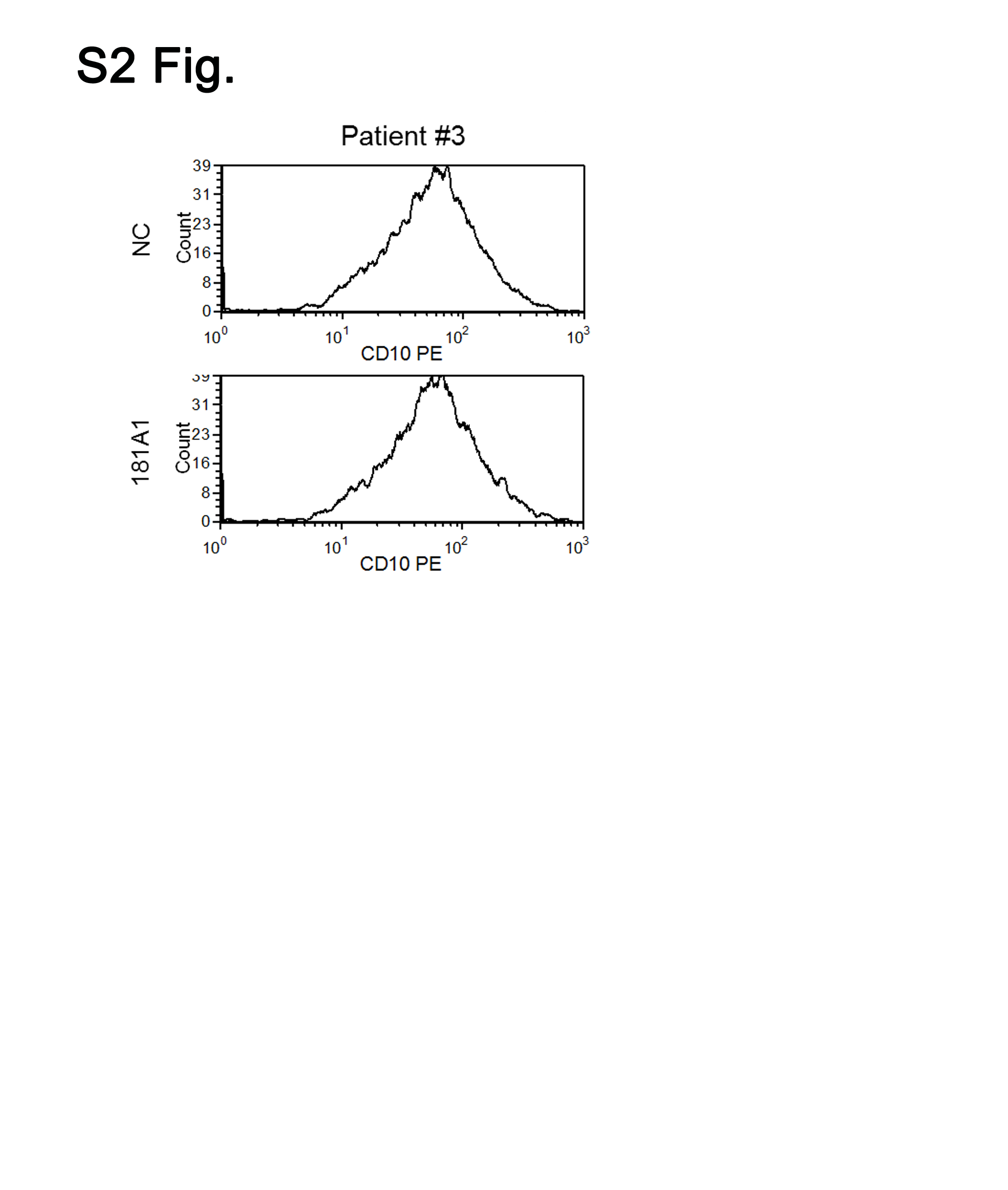

Supplement: S2 Fig — Flow cytometric analysis of CD10 expression on CD45w+or+ CD19+ ETV6/RUNX1-positive pre-B ALL blasts derived from patient #3. The MFI of CD10 in each groups were: NC 49.62, 181A1 51.68. (TIF) [file pone.0142863.s002.tif]

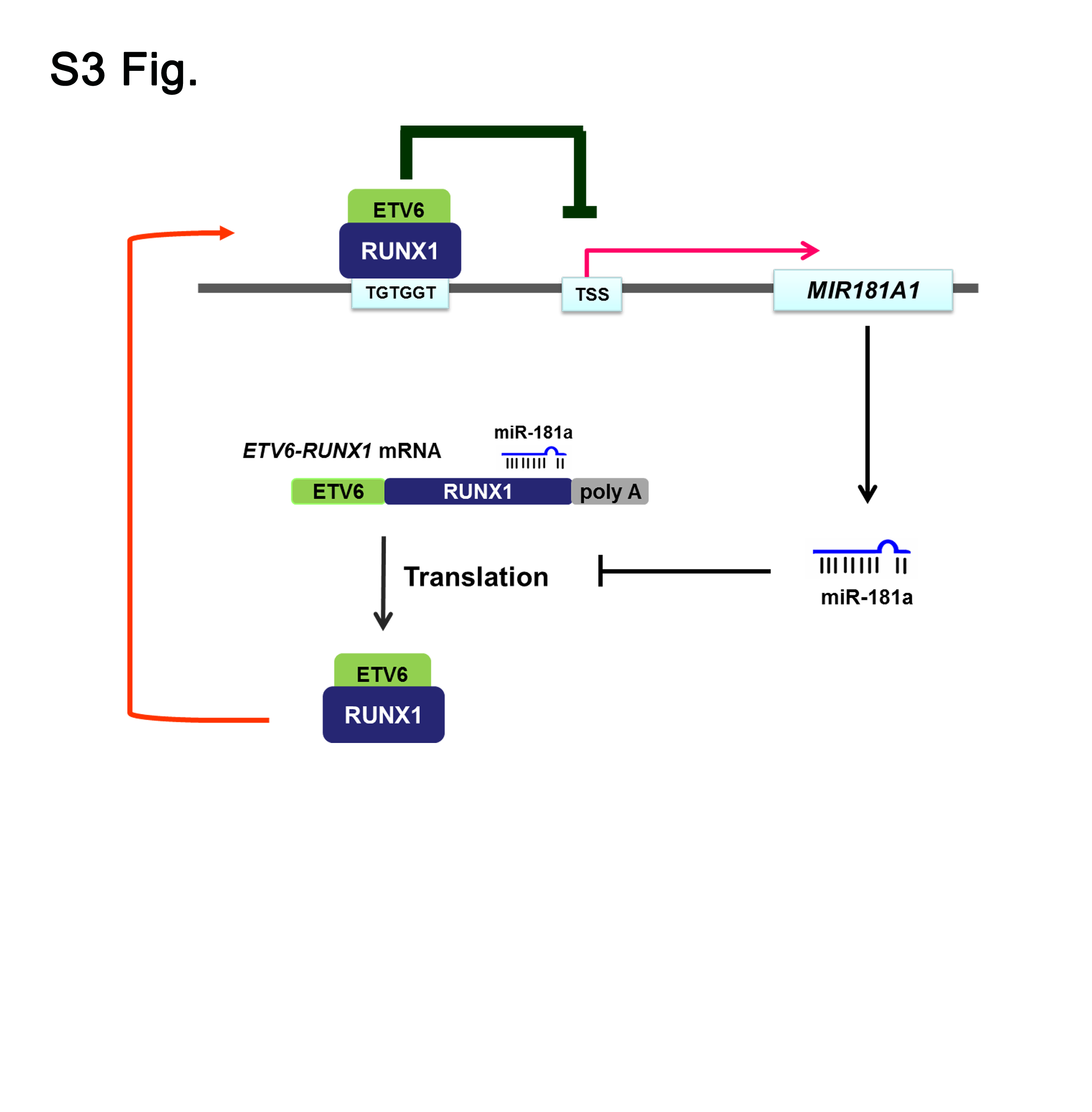

Supplement: S3 Fig — In leukemia cells with frequent chromosome rearrangement t(12;21)(p13;q22), ETV6/RUNX1 oncoprotein occupies the putative RUNX1-binding site upstream of MIR181A1 and restricts transcription by recruiting co-repressors such as HDAC3. This repression of MIR181A1 expression consequently upregulates the target of miR-181a, ETV6/RUNX1, the oncoprotein itself. (TIF) [file pone.0142863.s003.tif]
