# Supplementary material for: A Double Negative Loop Comprising ETV6/RUNX1 and MIR181A1 Contributes to Differentiation Block in t(12;21)-Positive Acute Lymphoblastic Leukemia
Source: PLoS One. 2015 Nov 18;10(11):e0142863. doi: 10.1371/journal.pone.0142863 (PMC4651427; doi:10.1371/journal.pone.0142863)
Supplement: S1 File — (DOCX) [file pone.0142863.s004.docx]

Supplements

1. Supplemental table：3 tables

| **Table A. Primer sequences** |  |
| --- | --- |
| **Primers for ChIP assays** | |
| P1-forward | 5'-CACCATACACAAACCACTTG -3' |
| P1-reverse | 5'-GAGCTCTGTGTATGATTGTC-3' |
| P2-forward | 5'-AG CTCAGTAGAGAGATGTTG-3' |
| P2-reverse | 5'-GGCACACAAGCTAAA ACTTG-3' |
| *GAPDH* coding region forward | 5'-GAAGGTGAAGGTCGGAGT-3' |
| *GAPDH* coding region reverse | 5'-ACCTTGAG CTCTCCTTGC-3' |

| **Table B. Clinical features of the ALL patients included in expression profiling study** | | | | | |
| --- | --- | --- | --- | --- | --- |
|  | ETV6/RUNX1^－^ | | ETV6/RUNX1^＋^ | |  |
|  | n | % | n | % | *P* |
| Gender |  | | | |  |
| Female | 20 | 50 | 4 | 40 | .728* |
| Male | 20 | 50 | 6 | 60 |  |
| Onset age |  | | | |  |
| Mean ± SD | 6.15 ± 3.23 | | 5.77 ± 2.97 | | .738† |
| Less than 10 | 36 | 90 | 9 | 90 | 1.000* |
| More than 10 | 4 | 10 | 1 | 10 |  |
| WBC count × k/μL |  | | | | |
| Less than 100 | 32 | 80 | 9 | 90 | .665* |
| More than 100 | 8 | 20 | 1 | 10 |  |
| t(9;22) |  | | | |  |
| Non | 37 | 92.5 | 10 | 100 | 1.000* |
| With | 3 | 7.5 | 0 | 0 |  |
| Risk groups |  | | | |  |
| SR | 20 | 50 | 6 | 60 | .899* |
| HR | 9 | 22.5 | 2 | 20 |  |
| VHR | 11 | 27.5 | 2 | 20 |  |
| SR: standard risk; HR: high risk; VHR: very high risk | | | |  |  |
| *Calculated by Fisher's Exact test | |  |  |  |  |
| †Calculated by Student's *t*-test | |  |  |  |  |
